# Differentiation of Motor Speech Disorders through the Seven Deviance Scores from MonPaGe-2.0.s

**DOI:** 10.3390/brainsci12111471

**Published:** 2022-10-29

**Authors:** Cécile Fougeron, Ina Kodrasi, Marina Laganaro

**Affiliations:** 1 Laboratoire de Phonétique et Phonologie, UMR7018 CNRS/Université Sorbonne-Nouvelle, 75005 Paris, France; 2 Signal Processing for Communication Group, Idiap Research Institute, 1920 Martigny, Switzerland; 3 Faculty of Psychology and Educational Science, University of Geneva, 1205 Geneva, Switzerland

**Keywords:** dysarthria, apraxia of speech, automatic classification, decision tree

## Abstract

For the clinical assessment of motor speech disorders (MSDs) in French, the MonPaGe-2.0.s protocol has been shown to be sensitive enough to diagnose mild MSD based on a combination of acoustic and perceptive scores. Here, we go a step further by investigating whether these scores—which capture deviance on intelligibility, articulation, voice, speech rate, maximum phonation time, prosody, diadochokinetic rate—contribute to the differential diagnosis of MSDs. To this aim, we trained decision trees for two-class automatic classification of different pairs of MSD subtypes based on seven deviance scores that are computed in MonPaGe-2.0.s against matched normative data. We included 60 speakers with mild to moderate MSD from six neuropathologies (amyotrophic lateral sclerosis, Wilson, Parkinson and Kennedy disease, spinocerebellar ataxia, post-stroke apraxia of speech). The two-class classifications relied mainly on deviance scores from four speech dimensions and predicted with over 85% accuracy the patient’s correct clinical category for ataxic, hypokinetic and flaccid dysarthria; classification of the other groups (apraxia of speech and mixed dysarthria) was slightly lower (79% to 82%). Although not perfect and only tested on small cohorts so far, the classification with deviance scores based on clinically informed features seems promising for MSD assessment and classification.

## 1. Introduction 

Motor speech disorders (MSDs) in adults can appear suddenly or progressively, depending on the underlying etiology (stroke or brain injury versus neurodegenerative diseases or brain tumors). Motor speech disorders include two main subtypes, apraxia of speech (AoS) and dysarthria, which are further subdivided into subtypes, at least for dysarthria. Despite the fact that AoS and dysarthria have been attributed to the disruption of different processes of motor speech planning/programming (impaired retrieving and/or assembling of speech motor plans in AoS [1,2,3,4,5], versus impaired motor programming and execution of the neuromuscular commands involved in speech production in dysarthria [3,6,7,8]), they share several clinical signs. For instance, slow speech rate and sound distortions are observed both in several subtypes of dysarthria and in AoS [6,7,8,9,10]. On the other hand, dysarthria can manifest through different patterns of impaired speech and several subtypes of dysarthria have been classically described in relationship to the impaired underlying pathophysiological neuro-subsystem [2,8]. Currently, at least seven subtypes are identified: flaccid dysarthria (in bulbar palsy), spastic dysarthria (in pseudobulbar palsy), ataxic dysarthria (in cerebellar disorders), hypokinetic dysarthria (in parkinsonism), hyperkinetic dysarthria (in dystonia and chorea), mixed dysarthria (e.g., a combination of flaccid and spastic dysarthria, as observed in amyotrophic lateral sclerosis). 

In clinical practice, the diagnosis of MSD and of its subtypes is mainly based on an auditory-perceptual approach in association with the information about the underlying neuropathology. The accuracy and inter-judge agreement of perceptual classification is nevertheless quite low [11,12,13,14,15,16], where even the two main subtypes (AoS versus dysarthria) are often confounded. For instance, in [16], speech and language therapists have often misclassified AoS and confused it with mixed dysarthria. As for the perceptual classification of subtypes of dysarthria, some subtypes seem to be easier to identify than others. For instance, hypokinetic dysarthria is often better identified perceptually than other types of dysarthria [15,16], while identification of “mixed” subtypes of dysarthria is quite bad [13,16]. These observations indicate that only some perceptual features of impaired speech are stable within a MSD category and/or easy to identify by ear, while most features or combinations of features of MSD are variable. On the other hand, low inter-judge agreement of perceptual classification is also inherent to the subjectivity of auditory-perceptual ratings that are based on the internal representations of each rater, or because some speech parameters are difficult to assess by ear. Given the difficulty of reliably describing impaired speech features/parameters with solely auditory-perceptual assessments, clinicians and researchers have sought acoustic approaches to describe impaired speech parameters and identify patterns of features leading to reliable classification of subtypes of MSD [17,18]. 

The MonPaGe-2.0.s protocol [19,20] provides a standardized assessment taking advantage of acoustic descriptors on several impaired speech parameters in MSD that are obtained via semi-automatic acoustic analysis routines, complemented by targeted/guided perceptual coding of some other speech aspects. The extracted acoustic and perceptual measures are compared to normative data, considering a matching in sex and age (from a dataset of 404 speakers), leading to a deviance score for each parameter. The MonPaGe-2.0.s screening protocol is currently based on a relatively limited set of seven descriptors linked to intelligibility, articulation, voice, speech rate, maximum phonation time, prosody and diadochokinetic rate measures. It has been shown to be quite performant in terms of sensitivity and specificity in detecting MSDs relative to neurotypical speech, even in the case of mild speech impairments [20]. Although the first diagnostic level when assessing speech is presence versus absence of MSD, a further step would be to tease out the different subtypes of MSD. Our aim here is to investigate whether the MonPaGe-2.0.s screening protocol provides the information on the impaired dimensions that allow the classification of different subtypes of patients with mild to moderate MSD. To this end, we will use the deviance scores from the different speech parameters/features assessed via the MonPaGe-2.0.s protocol to automatically classify speakers with MSD from six different neuropathological groups. 

If automatic classification (i.e. machine-based classification as opposed to human-based classification) exploiting the derived *deviance scores* (hereafter “DevS”) yields a high classification accuracy, it can be concluded that the MonPaGe-2.0.s DevS can be discriminative of the considered MSD subtypes. If automatic classification exploiting the derived deviance scores yields a low classification accuracy, alternative scores have to be sought for MSD differential diagnosis between our subgroups. To the best of our knowledge, the majority of automatic techniques in the state-of-the-art literature deal with classifying dysarthria against neurotypical speech, with most contributions considering hypokinetic and mixed dysarthria linked to Parkinson disease and amyotrophic lateral sclerosis respectively [21,22,23,24,25,26,27,28]. Automatic classification of subtypes of MSDs has only been seldomly considered, with the differentiation between apraxia of speech and dysarthria investigated in [29,30] and for instance the differentiation between dysarthria subtypes investigated in [31,32]. Further, state-of-the-art automatic techniques typically exploit a vast number of acoustic features extracted from the raw acoustic signal intended to capture impaired speech dimensions such as fundamental frequency, formant frequencies, jitter, shimmer, harmonics-to-noise ratio (HNR), Mel frequency cepstral coefficients or spectro-temporal sparsity [21,22,23,24,25,26,27,33]. In this paper, we investigate the automatic classification of several subtypes of MSDs. To this end, decision trees are trained as two-class classifiers to discriminate between each pair of MSD subtypes within the considered six different neuropathological groups. Instead of exploiting a vast number of acoustic features extracted automatically from the raw speech signals as in the state-of-the-art literature, we exploit only seven features, and these features are ‘clinically informed’, in the sense that the speech dimensions they capture are already expressed as ‘deviance’ from neurotypical speech.

## 2. Materials and Methods

### 2.1. Population

We included 60 patients with MSD, ten from each of six different neuropathology groups: two groups of patients with mixed dysarthria associated with amyotrophic lateral sclerosis (ALS) and Wilson disease; one group of patients with flaccid (bulbar) dysarthria associated with Kennedy disease; one group of patients with hypokinetic dysarthria associated with Parkinson Disease (PD); one group of patients with ataxic dysarthria in the context of spinocerebellar ataxia (SCA), and a group with apraxia of speech (AoS) following a left-hemisphere stroke. The data were extracted from the “MoSpeeDi dataset” (https://www.unige.ch/fapse/mospeedi/mospeedi-dataset accessed on 15 February 2021). The patients in the “MoSpeeDi dataset” were all French native speakers, had a neurological diagnosis established by neurologists in the hospitals of recruitment, and presented mild or moderate acquired or progressive speech difficulty noticed by the patient and a speech and language pathologist (SLP). No patients had a diagnosis of dementia or psychiatric disorder. The inclusion of the patients from the “MoSpeeDi dataset” was guided by the necessity to balance the severity of MSD across the six neuropathological subgroups. To avoid redundancy with the MonPaGe-2.0.s deviance scores used in the study, an external composite perceptive severity score was used as an inclusion criterion: the composite perceptive severity score (hereafter CPSS) from a French perceptive-based speech assessment protocol [34]. The CPSS score was computed via a perceptual rating on a 5-point scale (from normal - 0 - to severely impaired - 4 -) of the participant’s speech on five dimensions: voice quality, segmental realization, prosody, intelligibility and naturalness of speech (maximum score = 20). Two trained SLPs assessed each dimension on recordings of about two minutes of continuous read speech. Only patients with a minimum of 4 and a maximum of 14 in the CPSS score were included here, a range that is clinically associated with mild to moderate MSD. Five subgroups of patients partially overlapped with those in [20] (100% overlap for the groups of patients with post-stroke AoS and Wilson disease, and 90%, 60% and 20%, respectively, for the group with mixed dysarthria in ALS, with flaccid dysarthria in Kennedy disease and with hypokinetic dysarthria in PD. The distribution of the patients in the six clinical groups with associated basic descriptors and CPSS severity scores is presented in Table 1.

### 2.2. Assessment of Speech Dimensions

All the patients underwent speech assessment with the MonPaGe-2.0.s screening protocol (https://lpp.in2p3.fr/monpage/ accessed on 15 February 2021) in a clinical setting, with audio recorded with external sound cards and professional microphones (at a 44,100 Hz sampling rate). The MonPaGe-2.0.s protocol is based on semi-automated acoustic and perceptual measures on several speech dimensions in French and has been normalized on 404 neurotypical speakers aged 20–93 (from the MonPaGe_HA [19]) and validated on 80 speakers with MSD [20]. Two speech dimensions are evaluated perceptually (intelligibility and articulation) and five dimensions are assessed acoustically (maximum phonation time, voice, speech rate, prosody and diadochokinetic (DDK) rate). In the endeavor to define the set of deviance scores, our challenge was to determine which dimensions were adequate to accurately profile the MSDs, to differentiate MSD from neurotypical speech, and to differentiate MSD subtypes. In the vast array of potential speech features measurable [17], our selection was guided by the applicability of the protocol in clinical practice. In other terms, characterizing the recordings of the patients along the selected speech features needed to be little time- and expertise-consuming; to be weakly sensitive to the quality of the speech signal, both in terms of quality of the recording conditions but also in terms of the quality of the speech produced, which inherently deteriorated in MSDs (since both negatively impact the accuracy and validity of the measurement), and to be easy to extract. 

The tasks and measures for each speech dimension are briefly described below (for further details see [19,20]).

#### 2.2.1. Intelligibility

The intelligibility test is an interactive task between the examiner and the participant in a face-to-face setting, in which the participant has to instruct the examiner to place test-words on a 5 × 5 grid combining icons of various shapes and colors, using a pre-learned carrier sentence (“Place the word [target_word] on the [color] [shape]”, e.g., “Place the word [dog] on the [red] [circle]”). Fifteen test words are randomly drawn from a database of four hundred and thirty-seven picturable French words, with each word having several phonological competitors (minimal pairs). Only the participant sees the 15 test words on the computer screen and the examiner has to write each target word on a paper grid. The perceptual intelligibility score is computed off-line: it corresponds to the number of test words that are understood correctly by the examiner during the interaction.

#### 2.2.2. Articulation 

Articulatory precision was assessed on the production of a list of 50 pseudo-words (presented to the participants, both auditorily and visually, and in the same order), covering the articulation of most French consonants and vowels as well as consonant clusters. The scoring of articulation accuracy was also computed off-line based on the audio-recordings of the productions via a guided procedure that allows the raters to play each pseudo-word as many times as needed and score targeted phonemes or syllables as correctly or incorrectly pronounced. Overall, 151 target phonemes or syllables are assessed and the score is expressed in terms of a number of incorrect productions (from 0 to a maximum of 151).

#### 2.2.3. Maximum Phonation Time (MPT)

This is a standard measure of pneumo-phonatory control, based on maximum phonation time measured with a Praat [35] script over the sustained vowel /a/. The best performance out of two recorded trials is retained as a single measure.

#### 2.2.4. Voice 

Voice-related acoustic measures were based on a sustained production for 2-3 seconds of the vowel /a/ at a comfortable height and loudness and on the reading of a seven-syllable sentence composed of only voiced sounds (“Mélanie vend du lilas” (melanivãdylila), ‘Melanie sells lilac’). All measurements were computed with Praat using a semi-automatic procedure. Six measures related to voice were included: (a) over 2 seconds of the sustained /a/, we computed jitter (as the 5-point Period Perturbation Quotient), shimmer (as the 11-point Amplitude Perturbation Quotient), f0 standard deviation, and smoothed cepstral peak prominence (CPPs), (b) over the sentence, we computed f0 standard deviation and CPPs. A DevS was compounded for each measure, then combined in a unique composite voice deviance score (see below).

#### 2.2.5. Prosodic Contrast

The production of an assertive vs. interrogative prosodic contrast was tested on a four-syllable fully voiced sentence (‘Laurie l’a lu’ (loʁilaly), ‘Laurie has read it’) produced as a statement and as a question. The prosodic contrast between the two modalities was computed in terms of a difference in f0 modulation (f0 range in semitones) between the beginning and the end of the sentences measured with a guided Praat script. 

#### 2.2.6. Speech Rate

Speech rate was measured in phonemes/sec on the production of the short read sentence (“Mélanie vend du lilas” (melanivãdylila), ‘Melanie sells lilac’), with boundaries at sentence onset and offset determined with a guided Praat script.

#### 2.2.7. Diadochokinetic Rate

Maximum repetition rates with oral DDK tasks are often used in clinical practice to test the ability to make alternating articulatory movements in quick and accurate succession. Seven items, which vary in terms of phonological complexity, were used here. They included standard sequences used to compute alternating motion rate (AMR) with the repetition of a CV syllable (AMRCV) or a CCV syllable (AMRCCV). Different CV and CCV syllables were used to target alternating movements with different articulators: jaw/lips with /ba/, front part of the tongue with /de/, tongue body with /go/, back to front constrictions with /kla/ and front to back with /tʁa/. Finally, a repetitive sequence /badego/ was used to compute a sequential motion rate (SMRCV). Participants were instructed to produce these sequences in a continuous manner for at least five seconds as fast and as accurately as possible. An interval of four seconds was determined by a Praat script from a boundary placed at the onset of the production and was manually adjusted to the right in order to not cut the last syllable if needed. The number of phonemes produced over this interval was used as an index of DDK rate. In order to capture difficulties in the repetition of the same syllables (AMR) vs. the repetition of a sequence of three syllables (SMR) which could be found for speakers with AoS for instance [36], the difference between the sequential motion rate and the alternating motion rate averaged over all CV sequences was also computed (SMRCV - AMRCV). The four DDK scores (AMRCV, AMRCCV, SMRCV and SMRCV - AMRCV) were then combined in a unique composite DDK deviance score (see below).

### 2.3. Measures of Impaired Speech (Deviance Scores)

For each speech dimension, a deviance score (DevS) is calculated in MonPaGe-2.0.s in terms of deviance from the normative data according to the position of the speaker’s descriptor value relative to the corresponding (in age and sex) reference distribution. The DevS spans from no-deviance, i.e. within the normal range, to excessive deviance, with normal range defined as superior to centile 5, and severity of DevS computed based on centiles and inter-centile distance (DevS=1 : < c5 and ≥ c1; DevS=2: < c1 and ≥ 1.5*(C50-C5); DevS=3: >1.5*(C50-C5) and 2*(C50-C5); DevS=4 : > 2*(C50-C5)). For intelligibility, articulation, MPT, speech rate and prosody the maximum deviance score is 4 and relates directly to severity for each single measure. For voice and DDK rate, the DevS obtained on each of the six voice measures and on each of the four DDK measures are then combined in a composite DevS with a computation that leads to a maximum score of 6 in order to balance their weights when the DevS from the seven speech dimensions are summed for the total MonPaGe-2.0.s deviance score (totalDevS) (for more details of the computation of these composite DevS in MonPaGe-2.0.s see [20]). 

### 2.4. Automatic Classification

To investigate whether the MonPaGe-2.0.s screening protocol assesses impaired dimensions that allow discriminating between different subtypes of patients, we train vanilla decision trees for two-class automatic classification of the different pairs of MSD subtypes based on the obtained deviance scores. Decision trees are advantageous due to their interpretability, implicit feature selection and their ability to handle numerical and categorical data [30]. Fifteen different decision trees are trained to discriminate between AoS/Parkinson, AoS/ALS, AoS/Kennedy, AoS/Wilson, AoS/SCA, Parkinson/ALS, Parkinson/Kennedy, Parkinson/Wilson, Parkinson/SCA, ALS/Kennedy, ALS/Wilson, ALS/SCA, Kennedy/Wilson, Kennedy/SCA and Wilson/SCA. The quality of each split during training is measured through the Gini impurity [37], which measures the frequency at which any element of the dataset will be mislabeled when it is randomly labeled. Given the relatively small number of patients currently available in the corpus, validation is done following a leave-one-speaker-out validation strategy. To avoid overfitting, we set the maximum length of the longest path from the root to a leaf to three. Since we deal with small datasets and many two-class classifications, we decided not to tune this hyperparameter. Instead, we used the default value of three suggested in the used decision tree Python implementation [38]. The suitability of the MonPaGe-2.0.s DevS to discriminate between the different subtypes of MSDs is evaluated through the classification accuracy of the trained decision trees. Further, the importance of each DevS for each two-class classification is also analyzed. Feature importance refers to the usefulness of the feature at predicting the class of interest in each considered two-class classification. Note that feature importance is a direct outcome of the training process of decision trees and is computed as the total reduction of the Gini impurity in the training set brought by each feature.

## 3. Results

### 3.1. Description of Speech Profiles Per Group

**Figure 1:**
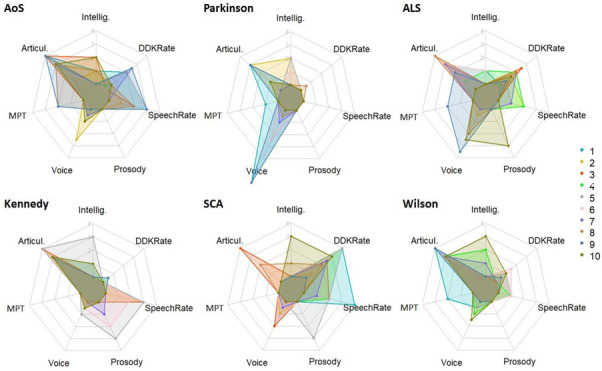
Individual DevS in each assessed speech dimension in each clinical group, from 0 to 4 for intelligibility, articulation, maximum phonation time (MPT), speech rate and prosody. For voice and diadochokinetic (DDK) rate the DevS is a composite computation of six and four DevS, respectively, with a maximum of 6. Articul: articulation; Intellig: Intelligibility; AoS: post-stroke apraxia of speech; ALS: mixed dysarthria in amyotrophic lateral sclerosis; Kennedy: flaccid dysarthria in Kennedy disease; SCA: ataxic dysarthria in spinocerebellar ataxia; Wilson: mixed dysarthria in Wilson disease.

Figure 1 presents the speech profiles of the ten speakers in each of the MSD subgroups included in the cohort, according to their deviance in each of the seven considered speech dimensions. In these radar plots, each color corresponds to a speaker and the scores are more deviant toward the periphery. Clearly apparent in these graphs is the variability of the impairments across speakers within each group, both in their degree of deviance (e.g., on the voice dimension for the PD group) and in the co-occurrence of features (e.g., in the group with Kennedy disease, deviance on prosody or speech rate co-occurs for two speakers with the other dimensions shared by the whole group). Shared deviant dimensions across groups are also clearly visible (e.g., deviance on the articulation dimension occurring in the AoS, ALS, Wilson and Kennedy group).

Nonetheless, some group profiles emerge such as the PD group with main abnormalities in articulation and voice, the Kennedy group with main abnormalities in articulation, the ALS and SCA groups with slow DDK rate, and the Wilson group with deviant articulation associated with impaired intelligibility. The AoS, ALS and SCA groups have a wider array of impaired dimensions than the other groups, extending to impaired DDK and speech rates associated with impaired articulation.

### 3.2. Two-Class Automatic Classification

Table 2 presents the overall classification accuracy for each two-class classification, i.e., the percentage of correctly classified patients out of the 20 patients belonging to the two classes. Further, the percentage of correctly classified patients from class 1 (out of the 10 patients in class 1) and the percentage of correctly classified patients from class 2 (out of the 10 patients in class 2) are also presented in parenthesis. 

The table is symmetric along the diagonal (except for the accuracies in parenthesis where class 1 and class 2 are interchanged). For each MSD subtype, the last row of the table presents the mean and standard deviation of all presented accuracies when discriminating between that subtype and the remaining subtypes. It should be noted that these average accuracy values cannot be interpreted as the performance one expects to obtain for the respective MSD subtype in a six-class classification setting. Instead, these average accuracy values can be used to compare which MSD subtype is on average easier to discriminate from the remaining subtypes in the considered two-class classification setting. Generally, it can be observed that the obtained automatic classification accuracies are relatively high (i.e., within the typical range that is considered to be high in the state-of-the-art literature on automatic classification between dysarthria and neurotypical speech, e.g. [21,24,26]), with the average performance ranging from 79% to 87%. Further, it can be observed that the best discrimination accuracy is observed for the SCA, PD and Kennedy groups (87%, 86% and 85%, respectively). The lowest average classification accuracy of 79% is obtained when discriminating between AoS and other MSD subtypes (dysarthria). However, the classification accuracies when discriminating between mixed dysarthria in ALS or Wilson disease and other MSD subtypes are also comparable, i.e., 81% and 82%, respectively. When considering individual two-class classification results, the best classification accuracy of 100% is obtained when discriminating between flaccid dysarthria in Kennedy and mixed dysarthria in ALS. Also, discriminating between AoS and ataxic dysarthria in patients with SCA or with hypokinetic dysarthria in PD and mixed dysarthria in Wilson disease yields a very high classification accuracy of 95%, meaning that out of 20 patients in each classification, only one is wrongly classified. More specifically, when discriminating between AoS and ataxic dysarthria in SCA, one patient with AoS gets misclassified as SCA. When discriminating between hypokinetic dysarthria in PD and mixed dysarthria in Wilson patients, one patient with PD gets misclassified as a patient with Wilson disease. Finally, the lowest classification accuracy when considering individual two-class classifications is 70%, which is obtained when discriminating between AoS and flaccid dysarthria in Kennedy disease or mixed dysarthria in ALS and ataxic dysarthria in SCA patients.

### 3.3. Feature Importance for all Considered Two-Class Classifications

**Figure 2:**
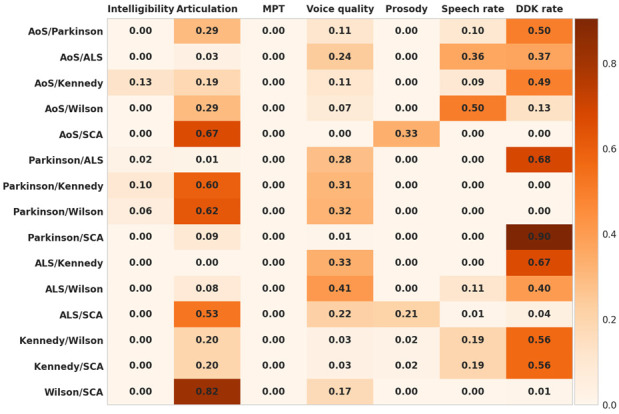
Importance of the seven DevS for every considered two-class classification. AoS: post-stroke apraxia of speech; ALS: mixed dysarthria in amyotrophic lateral sclerosis; Kennedy: flaccid dysarthria in Kennedy disease; SCA: ataxic dysarthria in spinocerebellar ataxia; Wilson: mixed dysarthria in Wilson disease.

Figure 2 presents the importance of each DevS for all considered two-class classifications. Feature importance is a scalar between 0 and 1, with a value of 0 showing that the feature is not useful for the classification and a value of 1 showing that the feature is the only useful feature for the classification. It should be noted that the importance values for all features in each classification (i.e., each row in Figure 2) sum to 1. Except for when discriminating between patients with AoS and patients with Wilson disease, either DDK rate or articulation are the most important discriminative features for all remaining two-class classifications. When discriminating between patients with AoS and patients with Wilson disease, speech rate plays the most important role. After DDK rate and articulation, voice quality is also important for many two-class classifications. The remaining DevS, i.e., intelligibility, MPT and prosody appear to be the least important, with MPT in particular not being useful in any of the two-class classifications.

## 4. Discussion 

As noted in the introduction, the main challenge in classifying patients with MSD relates to the facts that (a) deviant dimensions show considerable overlap between subtypes of MSD, (b) MSD profiles may be captured by the co-appearance or non-appearance of deviant features rather than single salient speech characteristics, and (c) large inter-speaker variation in speech profiles may occur within MSD subtypes [8]. All these aspects are clearly illustrated in the speech profiles of our population in Figure 1 on the seven speech dimensions selected in MonPaGe-2.0.s. Despite this well-known variability and overlap, the objective of the present work was to assess whether and to what extent the deviance scores from MonPaGe-2.0.s are able to differentiate major subgroups of MSD. 

In the remainder of this section, we discuss the performance of our DevS-based classification according to (1) its successes and the possible causes of its failures to differentiate MSD speakers, and (2) the contribution of each deviance score for differential diagnosis among MSDs. 

### 4.1. Success and Failures to Classify Subtypes of MSD 

Although the overall differential diagnosis based on the seven DevS is far from perfect, our results show that relatively high accuracy (ranging from 79% to 87%) can be obtained in determining a patient group membership over our pool of 60 patients, particularly considering the fact that the patients present only mild to moderate speech impairments. Overall, the two-class classifications predicted the patient’s correct category with 81% to 87% accuracy for the subgroups with dysarthria, while classification of the group with AoS was slightly lower with 79% accuracy. 

Interestingly, despite similarity in the severity of MSD across groups, the MonPaGe DevS appeared to better distinguish some MSD subgroups than others. This was the case for the groups with ataxic dysarthria in SCA, hypokinetic dysarthria in PD and flaccid dysarthria in Kennedy groups, which showed the highest classification accuracy rates.

The recognition of dysarthria as ataxic is valuable information for the localization of the neurologic dysfunction since ataxic dysarthria is usually associated with cerebellar disease (here spinocerebellar ataxia). Linked to impaired motor control, ataxic dysarthric speech is said to be predominantly poorly timed and discoordinated, although deficits may affect the different speech dimensions in a variable manner, resulting in a large variety of speech profiles [8,39]. This diversity is clearly visible in the heterogeneity of the speech profiles of our group with ataxic dysarthria in SCA. Nonetheless, the patients of this group are associated with the best classification scores: in all two-class classifications, at least eight out of the ten patients with SCA were correctly classified as belonging to the SCA group (100% and 90% in the SCA/AoS and SCA/PD classification, respectively, and 80% in the other classifications, see results in parenthesis in Table 2), and except for the patients with ALS (discussed below), no more than one patient of another group was wrongly classified as belonging to the SCA group. Two main features contribute to the correct classification of the patients with SCA: articulation, which is preserved for eight of the speakers with SCA as compared to the AoS, ALS and Wilson groups, and DDK rate, which is relatively slow (with a deviant score higher than two for eight of the speakers) as compared to the PD and Kennedy groups. While reduced speech rate is also a feature described for ataxic dysarthria in SCA [40], most of our speakers in the SCA group however did not present the impaired articulation observed in previous studies [40,41].

Hypokinetic dysarthria associated with PD has usually been shown to be quite well differentiated in perceptual classifications from other types of dysarthria (see introduction). Here, a good accuracy (90%) is also found for the classification of the patients with PD in all the two-class classifications of MSDs, except in the PD/ALS classification. In the PD/ALS classifications, three out of ten patients from the PD group were misclassified as ALS (while only one of the ALS was misclassified as PD). A similar error rate was found in a previous study testing perceptual classification on a set of patients overlapping in part the PD and ALS cohorts included here [16]: perceptually, the patients with hypokinetic dysarthria in PD were perceptually classified with only 72% accuracy in a PD/ALS classification. Analysis of the deviant dimensions contributing to the correct classification of patients with PD shows here that two different main features contribute the most to characterize hypokinetic dysarthria in PD: the absence of deficit in the DDK task (vs. presence in AoS, ALS and SCA), and quasi-absence (or weak) deviance on the articulatory dimension (vs. Kennedy and Wilson). 

Flaccid dysarthria associated with Kennedy disease can be misdiagnosed, at least at onset of the disease, as ALS (associated with mixed dysarthria with flaccid and spastic components). However, the differential diagnosis between the two dysarthria has strong implications for patient management [42,43]. Interestingly, our Kennedy/ALS classification allows us to predict group membership with 100% accuracy, with a strong contribution of the deviance in the DDK rate and voice, which are present only in the ALS group (probably linked to its spastic component). This absence of deviance in the DDK rate appears to contribute predominantly to the correct classification of speakers with Kennedy disease when compared to the groups marked by slow DDK rates (SCA, Wilson, ALS, AoS). Compared to the other MSD subtypes, dysarthria associated with Kennedy disease has been very little described in the literature. In previous studies, higher jitter, shimmer and HNR and hypernasality have been reported in only a subset of the patients with Kennedy disease [44,45]. In our data, no voice impairment was observed. On the other hand, all the patients with Kennedy disease had high deviance scores (3 or 4) on the pseudo-word production. These articulatory impairments could reflect reduced muscular force at the level of the tongue [46] or the jaw, as for patients with ALS. However, in the Kennedy group compared to the ALS group, articulatory distortions are not linked to a reduced speech rate, as also found in [43]. 

The mixed dysarthria in Wilson disease is said to combine features of ataxic, hypokinetic and spastic dysarthria, associated sometimes with hyperkinetic features [47]. Classification of the patients with Wilson disease was correct in most of the two-class classification (with 90 to 100% of the patients correctly classified as belonging to the Wilson group), except when tested with the Kennedy group. Indeed, in this Wilson/Kennedy classification, half of the patients with Wilson disease were misclassified as Kennedy. Further evidence for the failure in accurately classifying these patients is found in the feature contribution: the DDK rate DevS is the predominant feature used by the classifier, while little differences are found in this dimension between the two groups (only a few speakers with Wilson disease show some deviance in DDK rate in Figure 1). Another interesting aspect of the classifications involving the speakers with Wilson disease is that speakers with ALS and, to a lesser extent with SCA, were often misclassified as Wilson, probably due to the shared spastic and ataxic components. More surprisingly, 50% of the speakers with AoS were classified as Wilson, probably due to the absence of deviance in speech rate, which is more characteristic of the profile in the Wilson group and is variable in the profile of the group with AoS (see Figure 1).

In the ALS group, the involvement of both lower and upper motor neurons, with various prevalence, results in a mixed dysarthria with various dominance of flaccid and spastic components. This heterogeneity of speech profiles within the ALS group is reflected in our population (see Figure 1). Nonetheless, as previously mentioned, patients with ALS are never confused with the purely flaccid dysarthric patients of the Kennedy group. They are rather misclassified as belonging to the other mixed dysarthria with a spastic component (40% are classified as Wilson) or as belonging to the group with ataxic dysarthria (40% classified as SCA, a misclassification that has also been mentioned previously, [8]).

Finally, the poorest classification accuracy is found for the group with post-stroke AoS, except for the AoS/SCA classification. Apraxia of speech is rarely pure following stroke and the main challenge in the diagnosis of AoS is to distinguish AoS signs from possibly co-occurring dysarthria or aphasia. The speakers included in the post-stroke AoS group had dominant AoS signs but might also show concomitant mild non-fluent aphasia and/or associated dysarthria. This diversity in the AoS pool may be responsible for the heterogeneity in the speech profiles in this group, especially for the voice impairments that are rather associated with dysarthria. Speakers with AoS were mostly confused with Wilson (50%), PD (60%) and Kennedy (70%) groups in the relevant two-class classifications. One reason for these misclassifications is to be found in the shared impairment observed: deviant articulation (shared by the four groups), slow speech rate (shared with a few patients in the Kennedy group) and impaired voice quality (shared with a few patients in the PD group). Another reason for this poor classification of patients with AoS is that the deviance scores we use may not capture some of the salient features of AoS. Sound distortions, slow speech rate, distant substitutions, syllable segmentation, lengthened pauses, increased segment duration and groping are the seven top features for the diagnosis of AoS [48]. The DevS associated with articulation, speech rate and DDK rate may capture part of these aspects. However, they are obviously not sufficiently fine-grained to differentiate deviance on these dimensions linked to poor planning/programming from those linked to poor motor execution. Further development of the MonPaGe-2.0.s DevS scores is currently in progress to better capture different impairments linked to the unfolding of speech events in time and its variability.

### 4.2. Relative Contribution of the Seven DevS 

The deviance scores used in the MonPaGe-v2-0.s assessment protocol are grounded in offline perceptual judgments and in acoustic measures, which have been compared to normative references in order to define their deviance from neurotypical speech. In that sense, these deviance scores are ‘clinically informed features’. Therefore, a classification based on these deviance scores differs fundamentally from unsupervised automatic speech classification made on raw acoustic signals, as mentioned in the introduction. 

Based on the seven DevS, the MonPaGe-2.0.s screening protocol has already proven its sensitivity and specificity (see introduction and method). In the present study, we prove its potential applicability for differential diagnosis among most of the MSD subtypes examined and on a restricted number of speakers per group. However, examination of the feature importance for the different two-class classifications yields different feature contributions to the differential diagnosis. Indeed, out of the seven deviance scores, only four appear to really contribute to the two-class classifications.

We will discuss the features not contributing to the differential diagnosis first. 

The DevS Intelligibility showed almost no contribution to the classifications. This is not overtly surprising, since the population included in this study is weakly impaired on that dimension: only 19 out of the 60 speakers have a deviant intelligibility score larger than one and they are distributed over the six MSD subtypes. This is probably due to the fact that the population presents only mild or moderate MSDs.

The MPT score does not contribute to the differentiation of the MSDs either. Maximum phonation time is a classical task for the characterization of pneumo-phonatory control and is considered a non-speech task and a maximal performance task [49]. It is therefore not directly characterizing the speech of a patient, but may contribute to its clinical profile, since MPT can be reduced in the case of poor respiratory support as well as impaired phonatory function, which can occur in some MSD. However, this performance task, consisting in the production of a sustained /a/ vowel as long as possible, is inherently sensitive to intra- and inter-speaker variation [49,50]. In our normative data on 404 neurotypical speakers, MPT values did not show an age effect (consistent with previous reports [51,52]) and all speakers were merged into one single age group reference, with the consequences of lowering the average duration of MPT and of increasing the range of normal values. Therefore, the MPT score rarely reaches deviance level in the patients we have assessed so far with MonPaGe-2.0.s. Here, only one speaker with hypokinetic dysarthria in PD and two speakers with mixed dysarthria, one with ALS and one with Wilson, have a DevS indicating impaired MPT. In further development of the MonPaGe protocol, this feature may be discarded. 

The Prosody score developed in MonPaGe-2.0.s is aimed to capture the linguistic contrast between assertive and interrogative modality carried by f0 fluctuation. As such, it is restricted to a very specific aspect of linguistic prosody. In our population, only four speakers out of 60 present a deviance on this score. The mild severity of the patients already discussed could be an explanation, but the elicitation task itself (asking participants to transform an affirmative sentence into an interrogative one) may also be questioned. Further work may be needed to design a feature capturing other dysprosodic features linked to expressive or demarcative functions of prosody.

The four remaining scores are found to be much more informative in the differential diagnosis of our MSD subgroups. 

Oral DDK tests the ability to make alternating articulatory movements in quick succession. As such, it is a performance task [53] which cannot directly be related to speech-specific impairments according to some authors [54] (but see [55]). Nonetheless, DDK rates have proven efficient in distinguishing speakers with MSD versus neurotypical speakers (among many others: in PD [56], as an early manifest in Huntington disease [57], in ataxic dysarthria [58] and in AoS [55,59]). Our results show that the slow DDK rates contribute to a large extent to the classification of patients with AoS, mixed dysarthria in ALS and ataxic dysarthria in SCA versus patients with Kennedy disease, PD and Wilson disease. On the other hand, DDK also contributes to the classification of these latter MSD groups who have only minor DDK deviance scores. It should be recalled that the DDK score used here is a combined index based on the DDK rate for AMRCV, AMRCCV, SMRCV and the difference between the sequential motion rate and the alternating motion rate (SMRCV-AMRCV). Further work is needed to explore whether these components considered individually would improve the distinction among the MSDs showing altered DDK rates. 

Considering that slow, fast or irregular speech rate is one of the most commonly shared characteristics of many MSDs, it was not expected to have much discriminative power for differential diagnosis. In our population of mildly impaired patients, impaired (slow) speech rate was found for most of the patients with post-stroke AoS, ataxic dysarthria in SCA and mixed dysarthria in ALS (and two of the speakers with Kennedy disease). As found in [60], deviance in speech rate was not always related to deviance in DDK rate (see for instance the two patients in the Kennedy group who speak slowly but show normal DDK rate, or most of the patients in the ALS group who have slow DDK but normal speech rate). This brings a further argument to consider that these two dimensions need to be considered in combination for differential diagnosis of the MSD subgroups. Indeed, while DDK rate appears more informative than speech rate in most of the classifications, speech rate DevS has equal contribution to DDK rate for the AoS/ALS classifications, and is more informative than DDK rate for the AoS/Wilson classification. 

The Articulation score is the main contributor of the discrimination between the groups after DDK rate. It distinguishes the MSD groups with strong articulation disorders (AoS, SLA, Wilson and Kennedy) compared to groups with less impaired articulation (PD and SCA). As mentioned above, this feature could be refined to capture more subtle differences between MSDs in the type of sound distortion and particularly to capture complexity effects since the pseudowords used in MonPaGe-2.0.s are of varied complexities in terms of length and syllable structure. We expect to refine our characterization of AoS-specific patterns this way [61,62]. 

The voice quality score, which combines several features linked to alteration of voice quality, never ranks as the main contributor in our classifications except for the ALS/Wilson classification, where it is as informative as DDK rate. However, this feature does contribute to many of the two-class classifications: ALS vs. AoS, Kennedy, Wilson, SCA; PD vs. ALS, Kennedy, Wilson; Wilson vs. SCA. Components of this composite score will be individually explored in the future to investigate whether they provide a better discrimination of voice impairments in specific populations (for instance mono-pitch in PD group as captured by f0 standard deviation on the sentence). 

## 5. Conclusions

The use of automatic classification techniques has usually been applied to tease apart MSD from neurotypical speech; when applied to distinguish MSD subtypes, it is either restricted to a few subtypes and/or to a limited number of speech dimensions. Here, we show that discrimination between speakers from six subtypes of mild to moderate MSD is relatively performant with a set of seven features characterizing multiple speech dimensions, when these features are clinically informed in terms of deviance from neurotypical speech. 

Further directions to this work will involve seeking refinement of some of the present features in terms of granularity, and the addition of other features in order to improve the characterization of the fined-grained specificities of the speech impairments in the various MSDs considered and in other MSD subtypes. 

## Figures and Tables

**Table 1:** Patients’ clinical groups and associated descriptors: sex, age and CPSS.

Clinical group	Sex (F-M)	Age (range)	CPSS (range)
Post-stroke apraxia of speech (AoS)	6-4	52.5 (24–72)	8.5 (4–14)
Hypokinetic dysarthria in Parkinson Disease (PD)	2-8	74.5 (55–93)	7.2 (4–11)
Mixed dysarthria in amyotrophic lateral sclerosis (ALS)	3-7	61.5 (45–75)	8.6 (6–14)
Flaccid dysarthria in Kennedy disease	0-10	68.7(49–85)	7.7 (5–13)
Ataxic dysarthria in spinocerebellar ataxia (SCA)	7-3	50.4 (27–67)	7.8 (6–12)
Mixed dysarthria in Wilson disease	1-9	35.5 (26–49)	9.2 (5–14)

**Table 2:** Classification accuracy (i.e., percentage of correctly classified patients) for all considered two-class classifications. The first entry inside the parentheses corresponds to the percentage of correctly classified patients from class C1. The second entry inside the parentheses corresponds to the percentage of correctly classified patients from class C2.

		C1					
		AoS	Parkinson	ALS	Kennedy	Wilson	SCA
C2	AoS	-	75 (90/60)	80 (80/80)	70 (70/70)	75 (100/50)	95 (100/90)
	Parkinson	75 (60/90)	-	80 (90/70)	90 (90/90)	95 (100/90)	90 (90/90)
	ALS	80 (80/80)	80 (70/90)	-	100 (100/100)	75 (90/60)	70 (80/60)
	Kennedy	70 (70/70)	90 (90/90)	100 (100/100)	-	75 (50/100)	90 (80/100)
	Wilson	75 (50/100)	95 (90/100)	75 (60/90)	75 (100/50)	-	90 (80/100)
	SCA	95 (90/100)	90 (90/90)	70 (60/80)	90 (100/80)	90 (100/80)	-
	Mean ± SD	79.0 ± 8.6 (70.0 ± 14.1 / 88.0 ± 14.1)	86.0 ± 7.3 (86.0 ± 8.0 / 86.0 ± 13.6)	81.0 ± 10.2 (78.0 ± 16.0 / 84.0 ± 10.2)	85.0 ± 11.0 (92.0 ± 11.7 / 78.0 ± 17.2)	82.0 ± 8.7 (88.0 ± 19.4 / 76.0 ± 18.5)	87.0 ± 8.7 (86.0 ± 8.0 / 88.0 ± 4.7)

AoS: post-stroke apraxia of speech; ALS: mixed dysarthria in amyotrophic lateral sclerosis; Kennedy: flaccid dysarthria in Kennedy disease; SCA: ataxic dysarthria in spinocerebellar ataxia; Wilson: mixed dysarthria in Wilson disease.

## Data Availability

The data (the MonPaGe-2.0.s DevS) used in the study are available via the Yareta institutional open science archive at the following link: https://doi.org/10.26037/yareta:gypd5uhqgjgolb37kstuygf36m (accessed on 15 March 2021).
